# Supplementary material for: Predicting Mortality in Patients with Atrial Fibrillation and Obstructive Chronic Coronary Syndrome: The Bialystok Coronary Project
Source: J Clin Med. 2021 Oct 26;10(21):4949. doi: 10.3390/jcm10214949 (PMC8584483; doi:10.3390/jcm10214949)
Supplement: Supplementary file 1 [file jcm-10-04949-s001.zip › jcm-1432698-supplementary.pdf]

Supplementary materials

Table S1. Predictors of all-cause and cardiovascular mortality in all study participants using univariate analysis.

Variables	All-cause mortality		CV mortality	
	Hazard ratio (95% CI)	p	Hazard ratio (95% CI)	p
Age (for a 10 year increase)	1.79 (1.68–1.91)	<0.001	2.04 (1.87–2.22)	<0.001
Male	1.80 (1.57–2.03)	<0.001	1.76 (1.45–2.08)	<0.001
BMI (for a 10 kg/m^2 increase)	0.74 (0.66–0.82)	<0.001	0.82 (0.67–0.97)	0.01
Obstructive CCS	1.87 (1.65–2.1)	<0.001	2.27 (1.88–2.65)	<0.001
Double-vessel CAD	1.37 (1.12–1.62)	<0.001	1.54 (1.16–1.92)	<0.001
Multi-vessel CAD	1.58 (1.37–1.78)	<0.001	1.75 (1.44–2.06)	<0.001
Atrial fibrillation	2.44 (2.12–2.75)	<0.001	3.24 (2.68–3.79)	<0.001
Chronic heart failure	3.56 (3.12–4)	<0.001	4.68 (3.89–5.47)	<0.001
COPD	3.01 (2.40–3.63)	<0.001	2.99 (2.12–3.86)	<0.001
Diabetes mellitus	1.53 (1.33–1.73)	<0.001	1.48 (1.21–1.75)	<0.001
Chronic kidney disease	2.60 (2.28–2.92)	<0.001	2.92 (2.42–3.42)	<0.001
ASA at discharge	0.79 (0.68–0.91)	<0.001	0.68 (0.55–0.81)	<0.001
VKA at discharge	2.33 (1.99–2.66)	<0.001	2.94 (2.38–3.51)	<0.001
DOAC at discharge	1.44 (0.89–1.99)	0.03	1.87 (0.98–2.76)	0.002
ACEI/ARB at discharge	1.34 (1.05–1.62)	0.003	1.77 (1.17–2.38)	<0.001
BB at discharge	1.44 (1.1–1.78)	<0.001	1.66 (1.06–2.26)	<0.001
Statin at discharge	0.83 (0.7–0.96)	0.01	0.78 (0.61–0.95)	0.01
RBC (for a 10^6/mm^3 increase)	0.56 (0.49–0.63)	<0.001	0.56 (0.46–0.66)	<0.001
eGFR (for a 10 mL/min/1.73m^2 increase)	0.80 (0.78–0.83)	<0.001	0.79 (0.77–0.82)	<0.001
HDL (for a 10 mg/dL increase)	0.85 (0.82–0.89)	<0.001	0.85 (0.79–0.91)	<0.001

ACEI, angiotensin-converting-enzyme inhibitor; ARB, angiotensin receptor blocker; AF, atrial fibrillation; ASA, acetylsalicylic acid; BB, beta adrenergic receptor antagonist; BMI, body mass index; CAD, coronary artery disease; CI, confidence interval; CCS, chronic coronary syndrome; COPD, chronic obstructive pulmonary disease; CV, cardiovascular; DOAC, direct oral anticoagulant; eGFR, estimated glomerular filtration rate; HDL, high-density lipoprotein cholesterol; VKA, vitamin K antagonist; RBC, red blood cells.

Figure S1. Kaplan-Meier survival analysis of cardiovascular mortality in relation of the diagnosis of atrial fibrillation and chronic coronary syndromes.

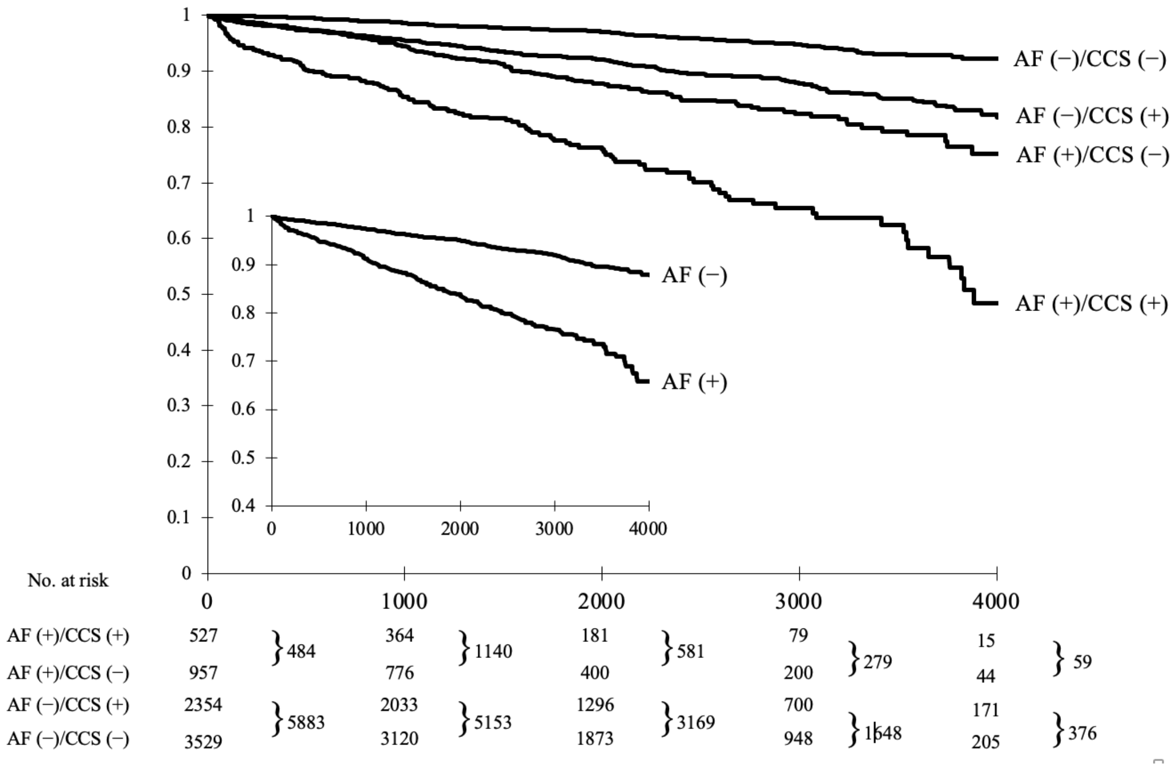

Abbreviations: AF, atrial fibrillation; CCS, chronic coronary syndrome

The inner graph represents the comparison between patients with and without atrial fibrillation independent of the diagnosis of obstructive chronic coronary syndrome.

All differences between curves are statistically significant (adjusted p values <0.01 for all tests).
